# *iCanADAPT Early* protocol: randomised controlled trial (RCT) of clinician supervised transdiagnostic internet-delivered cognitive behaviour therapy (iCBT) for depression and/or anxiety in early stage cancer survivors -vs- treatment as usual

**DOI:** 10.1186/s12885-017-3182-z

**Published:** 2017-03-15

**Authors:** M. J. Murphy, J. M. Newby, P. Butow, L. Kirsten, K. Allison, S. Loughnan, M. A. Price, J. Shaw, H. Shepherd, J. Smith, G. Andrews

**Affiliations:** 1 Clinical Research Unit for Anxiety and Depression (CRUfAD), UNSW School of Psychiatry at St Vincent’s Hospital, Level 4, O’Brien Centre, St Vincent’s Hospital, 394 Victoria Street, Sydney, NSW 2010 Australia; 2 School of Psychology, Faculty of Science, UNSW Australia, Mathews Building, Kensington, NSW 2052 Australia; 3 Nepean Cancer Care Centre, Sydney West Cancer Network, Kingswood, NSW 2747 Australia; 4 Psycho-oncology Co-operative Research Group (PoCoG), School of Psychology, Level 6, Chris O’Brien Lifehouse (C39Z), The University of Sydney, Sydney, NSW 2006 Australia

**Keywords:** Internet cognitive behavioural therapy, Depression, Anxiety, Cancer, Randomised controlled trial, Protocol

## Abstract

**Background:**

This RCT with two parallel arms will evaluate the efficacy of an internet-delivered transdiagnostic cognitive behavioural therapy (iCBT) intervention for the treatment of clinical depression and/or anxiety in early stage cancer survivors.

**Methods/design:**

Early stage cancer survivors will be recruited via the research arm of a not-for-profit clinical research unit and randomised to an intervention (iCBT) group or a ‘treatment as usual’ (TAU) control group. The minimum sample size for each group is 45 people (assuming effect size > 0.6, power of 80%, and alpha at .05), but 10% more will be recruited to account for attrition. A solitary or cumulative diagnosis(es) of Major Depressive Episode (current), Generalised Anxiety Disorder, Illness Anxiety Disorder, Panic Disorder, Agoraphobia, and/or Adjustment disorder will be determined using modules from the Anxiety Disorders Interview Schedule for DSM-5. Depression and anxiety levels with be measured via the total score of the Hospital Anxiety and Depression scale (HADS-T), the primary outcome measure. Secondary measures will include the Kessler 10 to measure general distress, the Fear of Cancer Recurrence Inventory (FCRI) to measure the specific fear of cancer recurrence and the Functional Assessment of Cancer Therapy, General Version 4 (FACT-G) for self-report of physical, social, emotional and functional well-being. iCBT participants will complete the measures before lessons 1 and 5, at post-treatment and at 3-month follow-up. The TAU group will complete similar measures at weeks 1, 8 and 16 of the waiting period. Program efficacy will be determined using intent-to-treat mixed models. Maintenance of gains will be assessed at 3-month follow-up. Mediation analyses using PROCESS will be used to examine the association between change in depressive and anxious symptoms over time and changes in FCRI and FACT-G QOL in separate analysis.

**Discussion:**

This is the first RCT looking at iCBT specifically for clinical depression and/or anxiety in a cancer population. Findings will help to direct the role of iCBT in streamlined psycho-social care pathways.

**Trial registration:**

Australian New Zealand Clinical Trials Registry: ACTRN12616000231448, registered 19^th^ February 2016 (www.anzctr.org.au). This trial protocol is in compliance with the Standard Protocol Items: Recommendations for Interventional Trials (SPIRIT) guidelines.

## Background

Emotional health problems are significantly higher in cancer survivors (defined as per the National Cancer Institute (NCI) definition, from the time of cancer diagnosis to the end of life) than in the general population. In a recent study, across the different types of cancer, 19% of patients had clinical levels of anxiety, and 12.9% having clinical levels of depression. In addition, other patients had subclinical levels of anxious (22.6%) and depressive (16.5%) symptoms [1]. Many cancer survivors suffer long-term emotional distress with symptoms of anxiety and depression often persisting for years following completion of cancer treatment [2]. There is a need for sustainable delivery of effective interventions for depression and anxiety in routine cancer care.

## CBT for the treatment of depression and anxiety in cancer

Fortunately there are clinically effective interventions available for the treatment of clinical depression and anxiety disorders in the cancer setting. A 2012 meta-analysis [3] concluded that Cognitive Behavioural Therapy (CBT) is an effective treatment for depression in cancer care while earlier studies found evidence for CBT in treating anxiety [4]. Additionally CBT in cancer care represents good value for money [5]. The barriers to accessing the clinically and cost-effective evidence based treatment of face-to-face CBT for depression and/or anxiety in the psycho-social context include: under-recognition of the problem by the patients, under-recognition of the need for screening and treatment of emotional health problems within teams and the lack of appropriately trained mental health professionals in many communities to provide evidence based care to cancer patients [6]. Leykin et al. [7] noted that too few cancer patients and survivors receive evidence-based interventions for psychological difficulties and that “*internet interventions have the potential to fill an important gap in quality cancer care by augmenting limited available mental health services*”. Furthermore, internet interventions overcome privacy and stigma concerns, allow access to therapy at a time and place convenient to the patient, including those living in rural and remote communities, and are reliable, cost-effective and reproducible [8–10].

## Online CBT for emotional health problems comorbid with physical illness

Online cognitive-behavioural interventions (iCBT) have been demonstrated to be effective for mild to severe anxiety and depression in the general population [11] and the overall effect-size is comparable to face-to-face CBT [12]. International interest is increasing in harnessing internet delivered interventions, such as iCBT, to tackle emotional health symptoms in patients with comorbid physical health conditions. Trials are underway to evaluate iCBT in treating comorbid emotional health symptoms in Cardiology [13, 14], HIV [15] and Diabetes [16].

There are numerous online programs, including some iCBT interventions, to assist cancer patients. A 2015 literature analysis identified *five* social support groups, *seven* online systems integrating information and *four* online therapies for psycho-social/physical symptoms [17]. Some programs are beginning to show results. In early stage cancer care, the ‘Finding My Way’ trial [18] provided preliminary support for the efficacy of an online intervention that teaches participants skills to adjust and cope after cancer diagnosis. Two further recent small studies have focused on the emotional health of cancer survivors. A recent Dutch RCT evaluated the ‘Cancer Aftercare Guide’, a self-management e-Health intervention [19, 20] which showed clinically relevant decreases of depression and fatigue in cancer survivors. An Australian feasibility study specifically for testicular cancer (eTC) survivors showed preliminary results in lowering depressive symptoms in a small group of survivors [21]. There are now larger trials underway looking at iCBT for severe fatigue in breast cancer survivors [22].

However, no intervention has been evaluated in a randomised controlled trial for a clinical depressive and/or anxiety disorder. A pilot program looking an CBT principles in a cancer survivor population with varying depressive symptoms [23] “*suggests that properly designed web-based interventions may be effective for reducing depressive symptoms among cancer patients*” and a Canadian feasibility open trial using a transdiagnostic iCBT intervention, in a group of 18 participants, showed the potential of using iCBT to decrease depressive and anxious symptoms in cancer survivors [24].

## ADAPT and development of iCanADAPT

The development of iCBT tailored to the cancer context and its integration into clinical care pathways is desirable at a system wide level to allow the integration of physical and emotional treatment. Clinical pathways provide evidence-based recommendations to guide best practice and consistent care for specific patient concerns in homogeneous patient groups. Clinical pathways are desirable at a system wide level to allow increased access to an appropriate level of interventions according to the severity of the patients’ symptoms [25]. ‘ADAPT’, or the ‘Anxiety and depression pathway program’, is a large multi-centred clustered implementation RCT funded by Cancer Institute NSW in 2015. ADAPT will evaluate different strategies of integrating and/or improving psycho-social care pathways in cancer care. One of the strategies included in the ADAPT program is the potential future use of iCBT as a treatment intervention. The ‘iCanADAPT Early’ iCBT program was developed as part of ADAPT to specifically address the needs of early stage cancer survivors, with a co-morbid clinical depressive and/or anxiety disorder.

The Clinical Research Unit for Anxiety and Depression (CRUfAD), UNSW at St Vincent’s Hospital, Sydney, has developed a number of iCBT programs for anxiety and depressive disorders including a transdiagnostic mixed anxiety and depressive disorder program for the general population [26]. CRUfAD modified this proven intervention and further specific cancer content was included. The further content was influenced by current evidence-based cancer-specific face to face CBT and a number of recently developed interventions in heterogeneous cancer patients (Finding my way [27], eTC [21]), Conquer Fear [28]). ‘iCanADAPT Early’ was iteratively reviewed by an expert panel comprising psycho-oncologists, psychiatrists, and consumers and revised accordingly. The final iCBT program is clinician supervised and delivered over a sixteen- week time period. The course is broken into eight lessons and tailored to the cancer context. The CBT skills incorporated facilitate the processing of cognitive and affective aspects of the cancer experiences and include stress management, problem solving, and other practical skills to manage symptoms.

Funded by the NSW Institute of Psychiatry, CRUfAD will conduct an RCT of the ‘iCanADAPT Early’ program, comparing the iCBT intervention to treatment as usual for early stage cancer survivors who meet criteria for a comorbid diagnosis of anxiety and/ or depression.

## Aims of ‘iCanADAPT Early’ study

- To examine the efficacy of a clinician-guided iCBT intervention in early stage cancer survivors experiencing anxiety and/or depression symptoms.
- To determine any concurrent changes in general distress, fear of cancer recurrence, and/or physical, social, emotional and functional status.
- To capture the feasibility of this iCBT treatment in terms of acceptability to patients.

## Hypotheses

The hypotheses are that, in a patient population characterised by comorbid early stage cancer survivorship AND a clinical depression and/or anxiety disorder:

1. iCBT plus treatment as usual will produce clinically relevant decreases in depressive and anxious symptoms as shown by a decrease in the total Hospital and Anxiety Depression Scale (HADS-T) score (primary outcome measure) compared to a control group receiving treatment as usual.
2. iCBT plus treatment as usual will produce clinically relevant decreases in general distress (Kessler 10), fear of cancer recurrence (Fear of Cancer Recurrence Inventory (FCRI)) and physical, social, emotional and functional status (as measured by the Functional Assessment of Cancer Therapy-General Version scale (FACT-G QOL)).
3. iCBT plus treatment as usual will be perceived by patients to be credible (Credibility/Expectancy Questionnaire(CEQ)), and to be likely to produce positive impacts.

The RCT was approved by the St. Vincent’s Hospital National Human Research Ethics Committees (reference: HREC/15/SVH/432).

## Methods/design

### Study design

This is a parallel group, randomised controlled trial comparing ‘Treatment as usual’ (TAU) plus up to 8 lessons of internet delivered CBT with clinician guidance’ (iCBT) against ‘Treatment as usual’ (TAU).

### Study setting

CRUfAD (Clinical Research Unit for Anxiety and Depression) is a non-profit joint initiative of St. Vincent’s Hospital, Sydney and the University of New South Wales, School of Psychiatry in Australia. CRUfAD specialises in the development, evaluation, and dissemination of evidence-based CBT programs via the internet. This RCT will be conducted within the Virtual Clinic, CRUfAD’s clinical research arm (www.virtualclinic.org.au). The mode of internet recruitment and delivery enables potential participants from all Australian states to apply for enrolment in the current trial.

### Recruitment

**Fig. 1:**
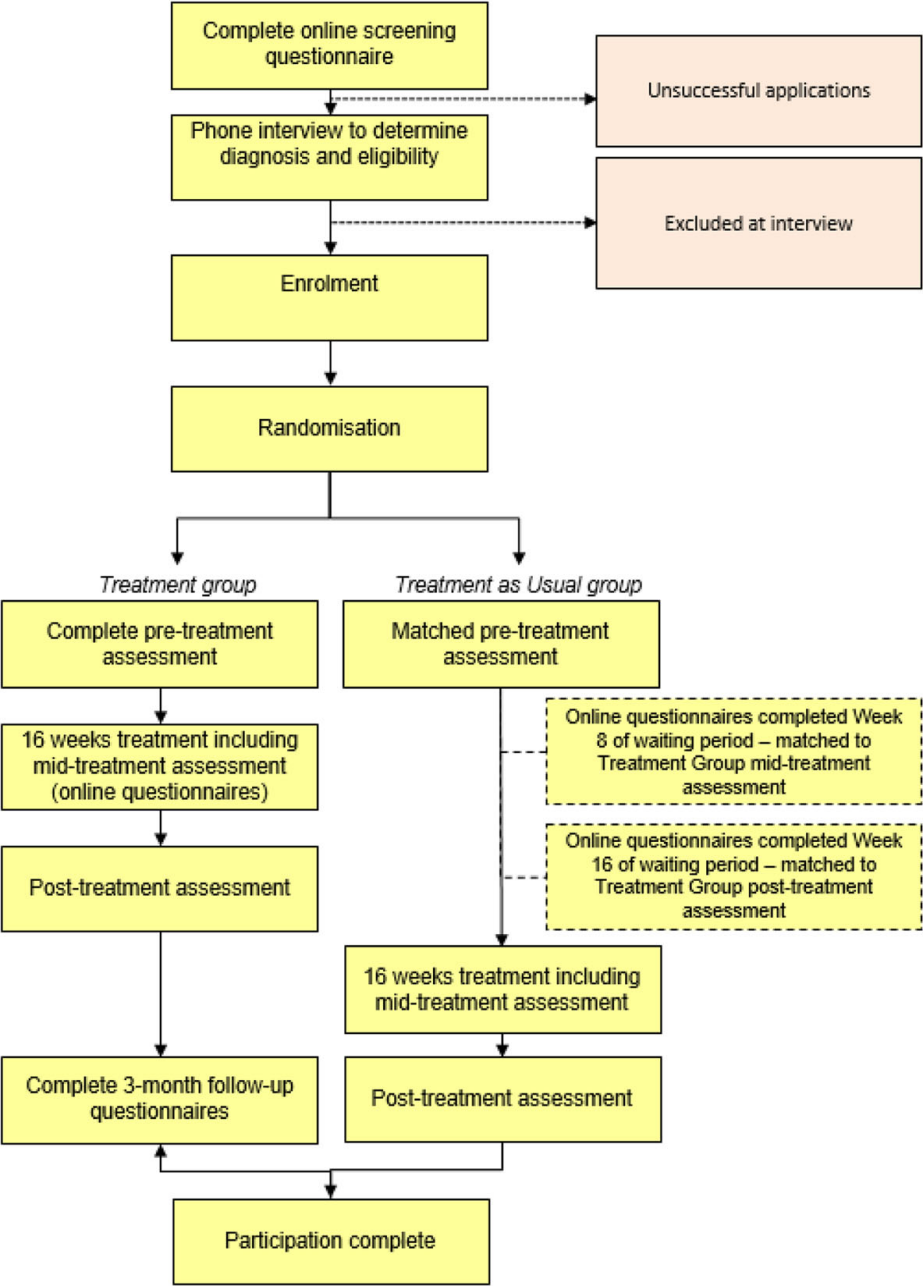
Participant Flow Chart

Participants will be recruited through flyers, internet social media advertising, cancer support groups and cancer research email listings. Applicants will first complete online screening questionaries about depressive and anxious symptoms and their demographic details will be collected. Inclusion criteria to proceed to the phone interview are as follows; resident of Australia; ≥ 18 years of age, a total score of ≥ 6 on the Hospital Anxiety and Depression scale (HADS-T) and a self-report of early stage cancer or cancer survivorship. This HADS-T cut-off was chosen as it will allow identification of 95% of cases [29, 30]. Applicants will also be asked a number of questions related to the exclusion criteria (see below) and will not proceed to phone interview if they endorse them. Applicants also complete the Alcohol Use Disorders Identification Test Consumption (AUDIT-C) questions but are not excluded on the basis of the results at this stage [31, 32]. See Fig. 1 for a participant flow chart.

### Inclusion/exclusion criteria for participation in the trial

All telephone interviews will be conducted by appropriately trained clinical staff. A psychiatry doctor will undertake the interview if one or more of the following are present: elevated HADS-T; any concerns of risk (elevated passive death wish/suicidality scores); high scores on AUDIT-C).

The inclusion criteria will be confirmed during the interview, namely: early stage cancer survivorship; access to a computer with internet and printer access; Australian residency; fluency in written and spoken English; willingness to provide name, phone number and address; and provision of name and contact details of their local general practitioner. The person will also be invited to identify their treating cancer centre. Upon confirmation of the above, the clinician will undertake the relevant modules from the Anxiety Disorders Interview Schedule for DSM-5. [33] for Major Depressive Episode (current) (MDD), Generalised Anxiety Disorder (GAD), Illness Anxiety Disorder (IAD), Panic Disorder (PD), Agoraphobia (Ag), and/or Adjustment disorder (Adj). The SCID Adjustment module will only be undertaken if the applicant has not met criteria for any of the preceding disorders.

*Cancer related exclusion* criteria will include any reported metastases, currently being an in-patient, being within the first 6 weeks of their cancer diagnosis, having a primary brain cancer (due to potential cognitive changes), a poor physical health status with an exclusion of an ECOG (Eastern Cooperative Oncology Group) performance status of greater than 2.

*Mental health exclusion* criteria will include currently receiving (i)CBT, or undertaking iCBT in the last year, a current or past history of schizophrenia or bipolar disorder, currently taking antipsychotic medications, frequent suicidality (indicated by a score of ≥ 2 to item 9 Beck Depression Inventory-II (BDI-II), or any concerns of active addiction on telephone interview.

At the time of telephone interview, any excluded applicants will receive information on alternative services and will be encouraged to discuss their symptoms with their physician.

Applicants who satisfy all inclusion criteria will complete an electronic informed consent prior to enrolling in the trial. Information from the diagnostic interview will be used for research purposes only for those participants who provide informed consent. All participants will be informed in writing that they may withdraw from the study (i.e. choose to cease program enrolment or choose for their data to be excluded from the RCT) at any time without jeopardising their relationship with St. Vincent’s Hospital or the University of New South Wales. Further, those participants randomised to the treatment as usual group will be informed that should they commence a CBT intervention or new psychotropic medication outside of the trial, their data will be omitted from the RCT.

### Trial design and blinding

The trial is a stratified randomised controlled superiority trial with parallel arms using a 1:1 allocation ratio. It is stratified according to ‘active treatment’ and ‘post treatment’. The ‘active treatment’ group are those who are at least six weeks from their initial cancer diagnosis, and are currently undertaking major cancer treatments such as chemotherapy or radiotherapy. The ‘post treatment’ group are those who have completed their primary treatment for cancer [34] with no upper timeframe. Experimenters and Participants will be aware of their group allocation but as all measures are self-report blinded assessors will not be required.

### Randomisation

Accepted participants will be stratified according to their phase in their cancer experience into the ‘active treatment’ group and ‘post-treatment’ group as described earlier. Thereafter, they will be randomised based on an allocation sequence generated by an independent person not involved in the study via a true randomisation process (www.random.org). Numbers corresponding to treatment group (1 = iCBT) or the treatment as usual group (2 = TAU) will be placed in sealed opaque envelopes bearing the sequential order number to ensure participant allocation based on the pre-determined randomisation sequence. A member of the research team will open the envelope after the diagnostic interview.

### Trial procedure

Figure 1: Participant Flow Chart details the time line for participants in the study. Once randomised, participants will be allocated to either the iCBT arm or the TAU arm. Over the sixteen week period, both groups undertake the same set of measures three times, the start, mid-point and end of the intervention. In the iCBT group, these times will vary according to when the participant undertakes the specific lesson; whereas for the TAU group, the questionnaires are according to specific times (i.e. weeks since commencement of group). Administration time points for measures are contained in Table 1. Baseline records of psychotropic medications and whether they are accessing psychological support will be captured for participants in both groups. Participants will be asked to inform the research team if any of their current interventions change.

**Table 1:** Administration time points for questionnaires used in the RCT

Measure	Pre-treatment^a^	Before each lesson^b^	Mid –treatment^c^	Post-treatment^d^	3-month follow-up^b^
HADS-T	♦		♦	♦	♦
K-10	♦	♦	♦	♦	♦
PHQ9-Q9	♦		♦	♦	♦
BDI-II Item 9	♦		♦	♦	♦
FCRI	♦		♦	♦	♦
FACT-G	♦		♦	♦	♦
CEQ	♦			♦	
ADIS	♦				♦

*HADS-T* Hospital Anxiety and Depression Scale,Total score, *K-10* Kessler-10 Psychological Distress Scale, *PHQ9-Q9* Patient Health Questionnaire 9-itme scale, Question 9, *BDI-II Item 9* Beck Depression Inventory-II, Item 9, *FCRI* Fear of Cancer Recurrence Inventory, *FACT-G* Functional Assessment of Cancer Therapy – General, *CEQ* Credibility and Expectancy/Satisfaction Questionnaire, *ADIS* Anxiety Disorders Interview Schedule for DSM-5

^a^iCBT group pre Lesson 1; TAU group at week 1 (Baseline)

^b^iCBT group only

^c^iCBT group pre Lesson 5; TAU group at week 8

^d^ iCBT group post Lesson 8; TAU group at week 16 (End)

### Description of the interventions

#### Internet delivered CBT (iCBT), the ‘iCanADAPT Early’ program group

The ‘iCanADAPT Early’ Program is a self-managed, 16 week online cognitive-behavioural intervention consisting of eight lessons involving general CBT skills and then cancer-specific CBT skills. Content is presented in the form of an illustrated comic-based storyboard in which two animated characters gain mastery over their anxiety and depressive symptoms with the help of a clinician. The female character is currently undergoing cancer treatment and tackling clinical levels of depression, while the male character is post-treatment with predominant anxiety symptoms. The participant follows both characters journeys to recovery across the 8 lessons. The *title*, and expansion of the main focus of each lesson are in Table 2: Course overview.

**Table 2:** Course overview

Lesson 1 - *Learning about Depression & Anxiety* Psycho-education about depression and anxiety, including their prevalence in cancer, psycho-education about the CBT cycle and the flight or fight response. Introduction to rumination and worry and good sleep hygiene. Skills of relaxation and progressive muscle relaxation. Lesson 2 - *Identifying and Tackling Thoughts* Key cognitive therapy skills including cognitive monitoring, recognising distorted cognitive styles, and later cognitive restructuring. Examples of each skill in use. Lesson 3 - *Tackling Unhelpful Behaviours* Key behavioural activation skills including tackling the low activity cycle, daily activity scheduling and the role of physical activity in improving general health. Additional skills relating to structured problem solving and assertive communication skills. Lesson 4 - *Helpful Coping* Further cognitive skills including worry-Free Zones and challenging mega-cognitions. Expanded attention to CBT strategies to tackle sleep difficulties. An introduction to mindfulness and related extra resources. Lesson 5 - *Adjusting to Change* Assessment of one’s personal values and goals. The need to pace physical activity. The use of CBT to tackle the impact of physical health (and/or treatment) symptoms on emotional wellbeing. Lesson 6 - *Tackling Avoidance* Introduction to avoidance and the rationale for combatting avoidance using graded exposure and exposure stepladders. Numerous examples of confronting avoidance (both in cancer and non-cancer settings) are intertwined to allow generalisation. Lesson 7 - *Mastering Your Skills* A guide to potential pitfalls that people encounter when trying to master CBT skills, in particular that of exposure work. An introduction to imaginal exposure. Lesson 8 - *Staying Well in the Long Term and Getting Even Better* Relapse prevention planning and an overview of the skills learned.	

At the end of each lesson, participants download a Lesson Summary which expands on the cartoon storyboard and provides a structured outline of the information. It also encourages participants to complete homework activities and review the lesson content at a later date. Extra resources are provided to participants and include worksheets examples.

## Clinician guidance for iCBT group

Each participant’s scores and engagement are monitored over the course of the working week. At minimum, individual email contact is made to each participant after the completion of Lesson 1 and Lesson 2. Thereafter, further email and/or phone contact will be made to participants depending on their needs. Typical reasons for clinician contact include an increase in distress scores, a participant question relating to content or a participant taking an unexplained break from logging in. Participants will have the opportunity to volunteer feedback about their most recent lesson/week(s) before each subsequent lesson and these responses will occasionally prompt clinician contact.

### Treatment as usual (TAU) group

Participants allocated to the TAU group may access their local general practitioner, community or cancer centre (mental) health services during the course of their waiting period.

## Clinician guidance for TAU group

Each participant’s questionnaire responses (week 1, 8 and 16) are reviewed. A generic email is sent from the Virtual Clinic system to thank participants for completing their questionnaire. Clinician contact will only be made if there are unexpected high HADS, or K-10 scores, or significant change from the scores previously obtained. Upon completion of the 16-week waiting period, and upon completion of the final set of TAU questions, the participants will transition to iCBT and then receive clinician guidance during their iCBT program as described above.

### Measures

#### Primary outcome measure

### Hospital anxiety and depression scale, total score (HADS-T)

The primary endpoint is anxiety and depression severity at post treatment as measured by the Hospital Anxiety and Depression Scale Total score (HADS-T) [35]. The HADS was developed as an outcome measure to assess anxiety and depression that avoids reliance on the measurement of somatic symptoms (e.g., fatigue, or insomnia) that may be experienced by people with cancer as part of the presenting illness. HADS-T is a fourteen item self-report questionnaire divided into two sub-scales, HADS-D, depression subscale and HADS-A, an anxiety subscale. The HADS-T is the total HADS score and is the combined score of the depressive and anxiety sub-scales. High scores indicate greater morbidity. This validated measure is widely used in clinical settings and has been validated in cancer patients [29].

### Secondary outcome measures

**Kessler-10 Psychological Distress Scale** (K10) [36]: The K10 contains 10 items ranked on a 5-point scale designed to measure non-specific psychological distress. High scores indicate higher distress. The K10 has been validated in a mixed cancer-type population [37]. Furthermore, the K10 is a quickly administered online tool and is advantageous for monitoring iCBT patients [38]. For the current study, the time frame was modified to assess psychological distress in the past 2 weeks rather than the past 30 days.

**Patient Health Questionnaire 9-item scale, Question 9** (PHQ9-Q9 [39]) **and Beck Depression Inventory-II, Item 9** (BDI-II,i9), [40]: The HADS-T and the K-10 do not contain questions regarding either a passive death wish or suicidal ideation. Therefore, these two screening questions for ‘better off dead’ thoughts and suicidality are included in the assessment of patients, and score changes are automatically reported. These questions allow the monitoring clinician to make contact with participants should changes occur.

**Fear of Cancer Recurrence Inventory** (FCRI) [41]: The 42-item Fear of Cancer Recurrence Inventory was chosen as it is an aggregate score of seven sub-scales ranging from triggers to level of insight into the problem. The FCRI severity subscale is reported to be the most informative regarding a patient’s level of fear [42]. High scores indicate greater fear of cancer recurrence. Possible associations between the FCRI subscale scores and the clinical diagnoses via the ADIS, and/or other determinants [43] including treatment response, will be assessed.

**Functional Assessment of Cancer Therapy – General** (FACT-G, version 4) [44]: The FACT-G is a 33-item general cancer quality-of-life measure. FACT-G has been established as a valid and reliable measure [45]. FACT-G was chosen over other quality of life measures as it has scales looking at the impact of symptoms on relationships and supports [46].

**Credibility and Expectancy/Satisfaction Questionnaire** (CEQ) [47]: At baseline, participants will complete two treatment expectancy questions: (1) “At this point, how logical does the program offered to you seem?” (0 = “Not at all logical”; 9 = “Very logical”) and (2) “At this point, how successfully do you think this treatment will be in reducing your depression and anxiety symptoms?” (0 = “Not at all useful”; 4 = “Very useful”). Following the combined intervention, participants will also rate treatment satisfaction: “How satisfied are you with the skills that this program has taught you to manage your depression and anxiety?” (1 = “Not at all satisfied”; 9 = “Very satisfied”); “How confident would you be in recommending this treatment to a friend who experiences similar problems?” (1 = “Not at all confident”; 9 = “Very confident”); followed by two questions about difficulty with the program and requesting additional feedback, which will require free text responses.

### Data management

All data will be collected via the Virtual Clinic software and stored on the Virtual Clinic server. All information collected will be coded with a participant identification number to allow data-to-patient matching. Additional clinical material not obtained via the online application, mainly pertaining to data obtained via the diagnostic intake interview, will be collected and stored in written format in a secure location in CRUfAD. Any identifiable information will remain confidential, except as required by law. Only members of the site research team will have access to participant information and data in order to monitor patient progress. During data analysis, re-identifiable data (i.e. coded data) will be used. At study completion, non-identifiable data will be written to a password-protected database. All data will be extracted from the Virtual Clinic servers. Analysis will be conducted using Statistical Package for the Social Sciences (IBM SPSS, IBM Corp., Armonk, NY, USA).

Participants will be informed in writing that the research team plans to disseminate the trial results in peer-reviewed scientific publications and presentations. Participants are informed that in any such dissemination, their anonymity will be maintained. Participants will be sent (via email) a written summary of the results in lay terms following completion of the trial study phase.

### Statistical methods

There is no prior published research outlining effectiveness of iCBT for treatment of a clinical depression or anxiety disorder in a cancer setting. In order to calculate sample size, data was taken from published randomised controlled trial of a transdiagnostic iCBT program [26]. Assuming the effect size of >0.6, and taking power at 80% with alpha set at 0.05%, sample size is calculated to be 44 per group. This is to detect a 0.6 effect size for the iCBT group to improve more than the treatment as usual group. Recruitment of 50 per group will be undertaken to account for attrition.

All analyses will be conducted at conclusion of the trial period, i.e. when all participants in the treatment group have completed the iCBT program. All analyses will be implemented in SPSS. Consideration will be given to the stratification into ‘active treatment’ and ‘post-treatment’.

Significance testing of group differences regarding demographic data and pre-treatment measurements will be conducted using analysis of variance (ANOVA) and *χ*2 where the variables consist of nominal (or categorical) data. Intent-to-treat (ITT) mixed models using restricted maximum likelihood (REML) estimation will be used to account for missing data due to participant drop-out. Mixed models do not assume that the last measurement is stable (the last observation carried forward assumption) [48, 49]. REML models are appropriate for RCTs with multiple time points and pre-to post-only designs [50]. The assumption that data are missing at random (MAR) will be evaluated using binary logistic regression to predict drop-out (0 = no drop-out, 1 = drop-out) and by comparing these two groups on baseline measures. Significant effects will be followed up with pairwise contrasts comparing mean pre-treatment to mean post-treatment scores. Mediation analyses will be used to examine the association between change in depression and anxiety symptoms over treatment and change in each of FCRI and FACT-G in separate analyses. Tests of the indirect effects (mediation) will be conducted using PROCESS [51].

## Discussion

This RCT will evaluate the efficacy of an iCBT intervention for clinical depression and/or anxiety in people with early stage cancer going through treatment or post-treatment. As the majority of e-health interventions in psycho-oncology to date focus on supportive care [17], this is a novel program as it will evaluate iCBT in clinical depression and/or anxiety. Fortunately, cancer survivors have an overall “positive attitude” to self-management and e-Health [52].

If proven efficacious, and acceptable to patients, the iCBT intervention would be added to the armoury of psychological treatments offered to cancer survivors suffering from anxiety and depression symptoms generally. It will be a core component of the forthcoming ADAPT Cluster Randomized Controlled trial. Moorey [53] importantly asked in 2013 “*I know they are distressed, what do I do now?”.*, He outlines that, despite many advances in Psycho-Oncology, services need to incorporate less labour intensive methods of delivery of therapy (primarily CBT), in order to be widely accessible and sustainable. He proposed that internet therapy will have a role in the changing interface of how care is provided in the cancer setting. This trial will add to the research aiming to address the short fall he outlines. It will add to recent work on the role of online interventions in other areas of supportive care, such as fatigue and acute distress [54], for people living with, or survivors of cancer. As an RCT aiming to recruit over 100 participants, it will add to the literature base and build on the pilot and feasibility studies in this area of psycho-oncology. Furthermore this trial will add to the growing literature of the use of iCBT for treating emotional health problems in patients with a co-morbid physical illness.

Online, or internet, interventions are easy to access, including in rural areas, accessible by the user at any time, private and confidential, self-empowering, scalable and cost-effective and can meet the needs of most patients, freeing staff to see only the most severe cases [11]. As Mitchell [55] pointed out “*screening is generally ineffective without aftercare*”; hopefully iCBT can become a viable, evidence-based treatment option in the future, thereby increasing treatment afforded to patients screening positive for distress and depression.

### Trial status

Trial registration: Australian New Zealand Clinical Trials Registry: ACTRN12616000231448, registered 19^th^ February 2016 (www.anzctr.org.au). This trial protocol is in compliance with the Standard Protocol Items: Recommendations for Interventional Trials (SPIRIT) guidelines. The first patient was enrolled 01^st^ March 2016. Data analysis is scheduled for late 2017.
